# Guided Endodontics as a Minimally Invasive Approach for Calcified Root Canal Management: A Case Report

**DOI:** 10.7759/cureus.93253

**Published:** 2025-09-26

**Authors:** Gargee Kasar, Nandan Rao K, Amit Padmai, Prajakta Swami, Wineet Magdum

**Affiliations:** 1 Department of Conservative Dentistry and Endodontics, Tatyasaheb Kore Dental College and Research Centre, Kolhapur, IND

**Keywords:** dental pulp calcification, guided endodontics, minimal invasive dentistry, nonvital tooth, root canal therapy

## Abstract

Calcified root canals present a significant clinical challenge due to reduced canal visibility, increased risk of iatrogenic damage, and limited access to the apical third. Conventional methods often compromise tooth structure while attempting to locate the obliterated canals. Guided endodontics, integrating cone-beam computed tomography (CBCT) imaging and three-dimensional (3D) printing, offers a minimally invasive and precise alternative for managing such cases. This case report describes the endodontic management of a tooth with severe pulp canal obliteration using a digitally planned and 3D-printed guide to facilitate canal localization and instrumentation. A CBCT scan was used to identify the remaining canal pathway, and a customized access guide was fabricated to ensure conservative preparation and accurate entry. Treatment resulted in successful canal negotiation, cleaning, shaping, and obturation without procedural complications. This case highlights the clinical efficacy of guided endodontics in overcoming anatomical challenges, preserving tooth structure, and improving treatment outcomes in calcified root canal cases.

## Introduction

Pulp canal calcification is a common consequence of various local and systemic factors, including dental trauma, age-related physiological changes, and the deposition of reactionary or reparative dentin [1]. Following traumatic injuries, particularly luxation, canal obliteration has been reported in 9-40% of cases [2]. Calcific changes may also arise secondary to restorative procedures, cervical pulpotomy, or prolonged orthodontic forces, all of which stimulate dentin deposition within the root canal space [1]. Clinically, such teeth are often asymptomatic and detected incidentally on radiographs, but they may present with discoloration or progress to pulp necrosis and apical pathology, necessitating endodontic treatment [3].

Management of calcified teeth poses a significant clinical challenge. The reduced or absent pulp chamber complicates canal identification and negotiation, increasing the risk of perforation, excessive dentin removal, or deviation from the natural canal trajectory [4]. Conventional techniques, even when performed under magnification and by experienced operators, may be unpredictable and can compromise long-term prognosis.

Guided endodontics has emerged as a minimally invasive and highly accurate alternative for accessing calcified canals. This technique combines cone-beam computed tomography (CBCT), intraoral scanning, and computer-aided design/computer-aided manufacturing (CAD/CAM) to create a three-dimensional (3D)-printed guide that directs the access cavity precisely to the canal orifice [5,6]. Unlike conventional methods, guided endodontics ensures controlled and predictable access, reducing the risk of procedural errors. Once the access is established, standard biomechanical preparation and obturation can be performed safely. By providing this brief overview, the introduction aims to make the concept of guided endodontics accessible to readers who may be less familiar with digital or template-guided approaches, including postgraduate students and clinicians with limited experience in advanced endodontic technologies.

## Case presentation

Patient information

A 45-year-old female patient reported to the Department of Conservative Dentistry and Endodontics with the chief complaint of pain and discoloration in the maxillary anterior region for the past few months. The pain was described as mild, gradual in onset, and persistent. The patient’s history revealed trauma due to a fall 10 years earlier, followed by avulsion of tooth 21. No relevant medical history was reported. Informed consent was obtained from the patient, and guided endodontic treatment was planned following confirmation of root canal calcification and endodontic pathology through clinical examination, radiographs, and CBCT assessment.

Clinical findings

**Figure 1:**
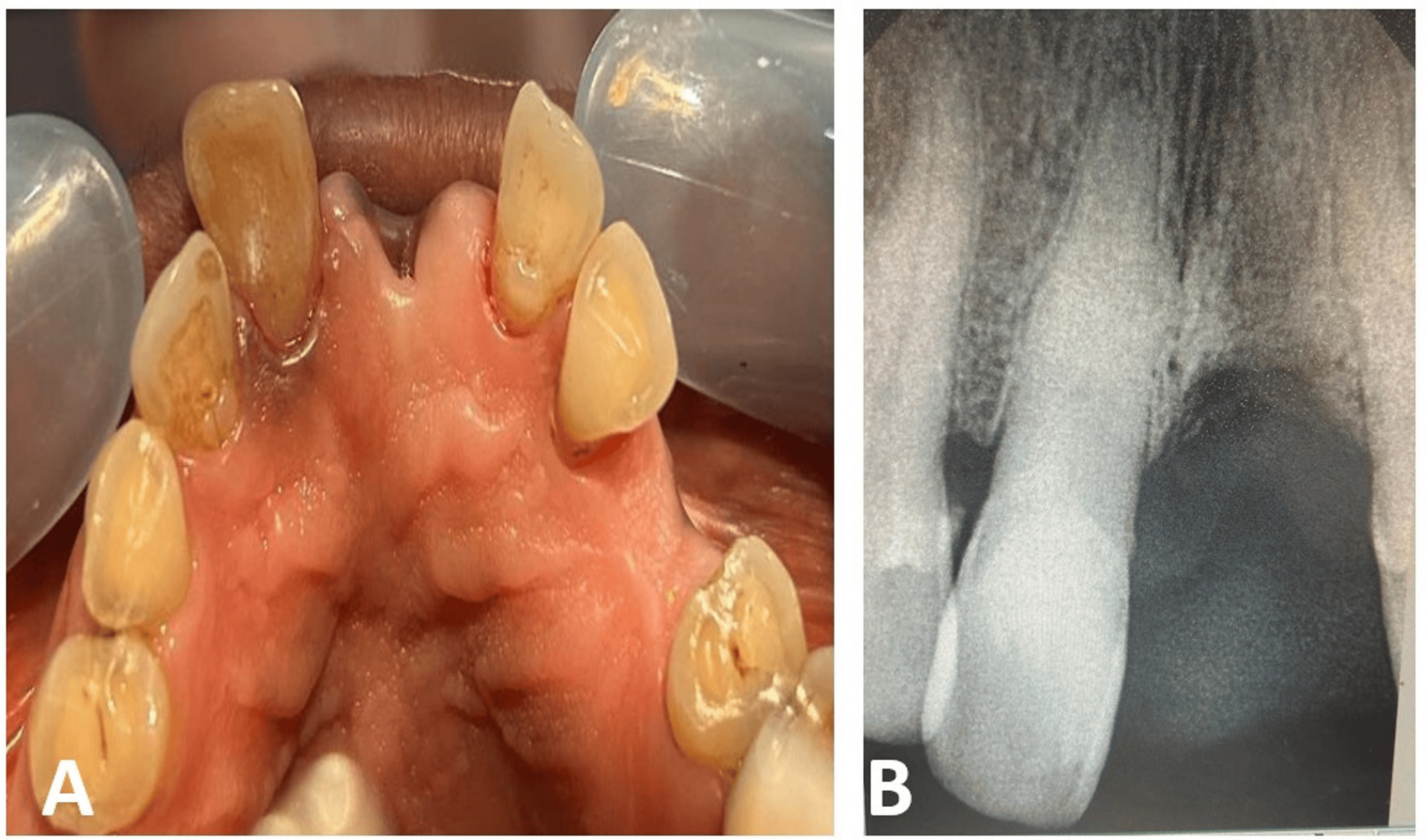
Preoperative images (A) Intra-oral maxillary arch showing tooth discoloration in tooth 11; (B) Intra-oral periapical radiograph (IOPAR) showing the degree of calcification in the root canal and periapical lesion in tooth 11

Clinical examination revealed discoloration of tooth 11 (Figure 1A). The tooth exhibited mild tenderness on percussion, but no mobility, swelling, sinus tract, or periodontal pockets were detected. Pulp sensibility testing using an electric pulp tester elicited no response. Intraoral periapical radiograph (IOPAR) revealed calcific obliteration of the pulp chamber, a periapical radiolucency, and early signs of external resorption (Figure 1B).

Diagnostic assessment

For further evaluation, limited field-of-view CBCT imaging was performed using a Planmeca Romexis scanner (5.3.5.80, G-XR-136953, Helsinki, Finland) at 90 kV and 10 mA. Coronal, axial, and sagittal sections confirmed pulp canal obliteration and apical changes in tooth 11. The Digital Imaging and Communications in Medicine (DICOM) files obtained from CBCT were processed for guided access planning.

Therapeutic intervention

Digital Planning Workflow

**Figure 2:**
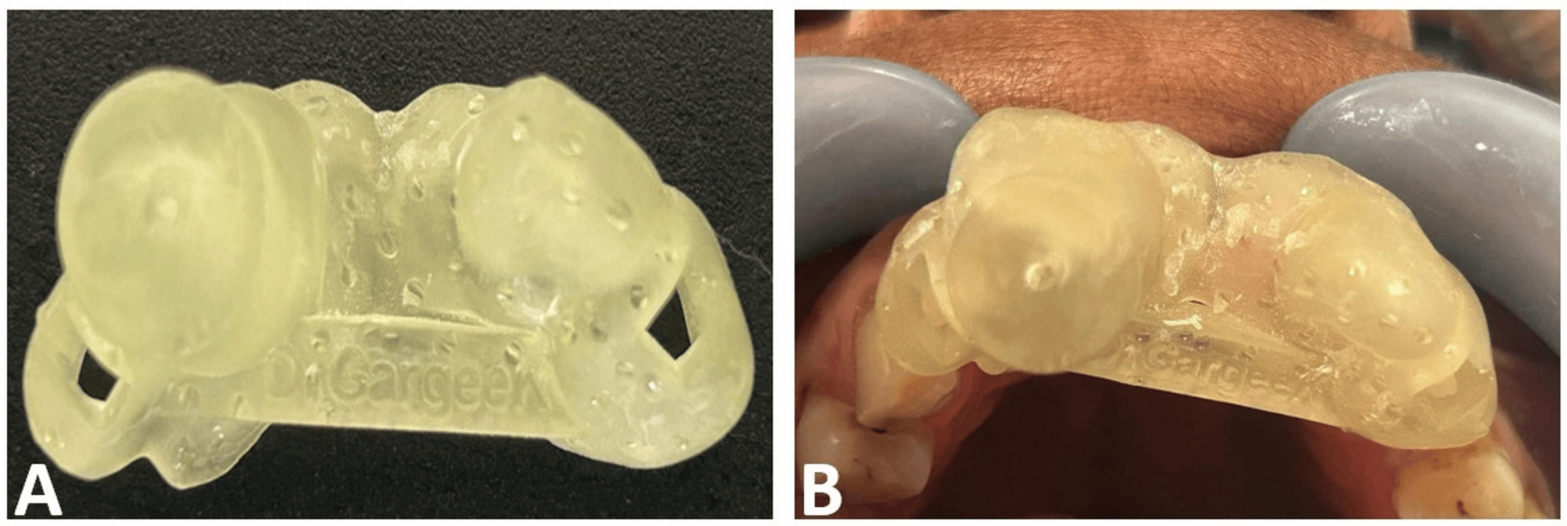
Guided stent fabrication (A) Ex-situ inspection of the guided stent to check the structural durability; (B) In-situ inspection of guided stent to check for fit

The CBCT data were converted into a stereolithography (STL) file using 3D Slicer software ver. 5.0.3 R30893/7ea0f43 (https://www.slicer.org/). These STL files were imported into 3Shape Implant Studio (3Shape, Copenhagen, Denmark), where a virtual guide rod was designed and aligned with the anticipated canal trajectory. The finalized design was exported, oriented on the print bed, and fabricated using a photocurable resin 3D printer (Ackuretta DENTIQ-120; Ackuretta Technologies, Taipei, Taiwan) (Figure 2A). The stent was then inspected and verified intraorally for accurate fit (Figure 2B).

Clinical procedure

Local anesthesia was administered using 2% lignocaine with adrenaline (Lignox 2% A; Indoco Remedies Ltd., Mumbai, India) by infiltration technique. The customized stent guide was seated on the maxillary anterior teeth, and its fit was verified. Caries-detecting dye (Waldent Innovations Pvt. Ltd., New Delhi, India) was used to confirm the access entry point in tooth 11. Following this, a rubber dam was used for isolation.

**Figure 3:**
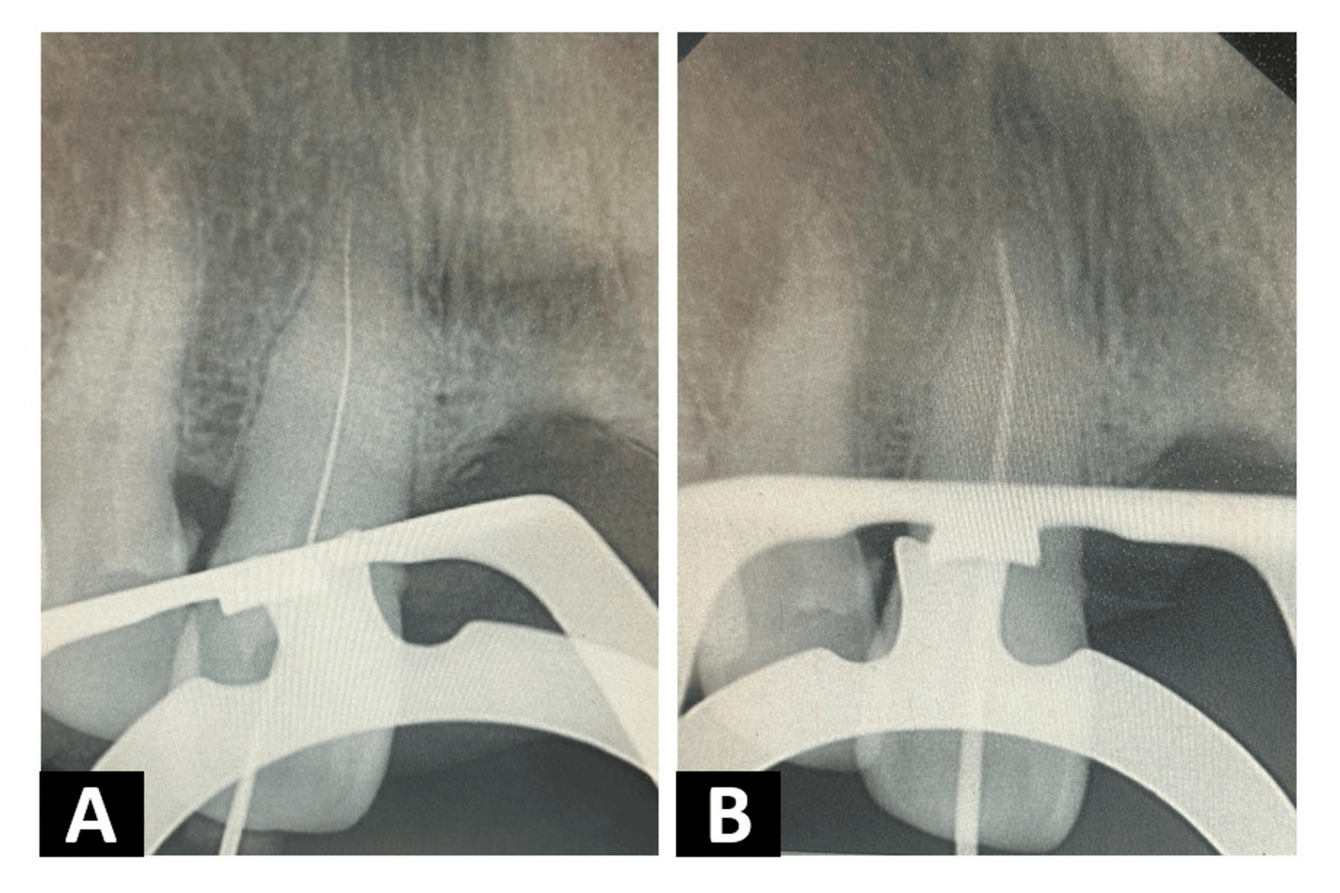
Intra-oral periapical radiograph (A) Working length determination; (B) Master apical cone

A surgical bur (Mani Inc.; Tochigi, Japan) was advanced through the stent sleeve until the calcified pulp chamber was reached. A size #08 K-file (Mani Inc.) was introduced along the guided path, and working length was determined using an electronic apex locator (Root ZX; J. Morita Mfg. Corp., Kyoto, Japan) and confirmed radiographically (Figure 3A).

A 25 mm K-file was utilized, and the working length was established at 22 mm. All biomechanical preparations were performed without the stent in place. The customized stent was employed solely during the access cavity preparation, wherein only a long-shank bur was used to negotiate the calcified entry path.

Cleaning and shaping of the canal were performed using standardized circumferential filing with copious irrigation of 3% sodium hypochlorite (Prime Dental Products Pvt. Ltd., Thane, India) and 15% ethylenediaminetetraacetic acid (EDTA) (RC Help; Prime Dental Products Pvt. Ltd.). Apical enlargement was achieved up to a size #40 K-file, and a corresponding #40 master cone was verified radiographically (Figure 3B).

**Figure 4:**
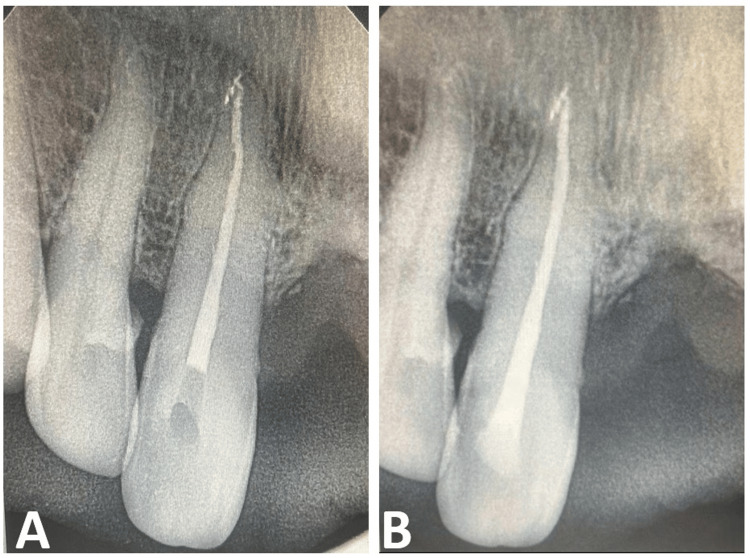
Postoperative intra-oral periapical radiograph (A) Obturation; (B) Post-endo restoration

The canal was obturated using the lateral compaction technique with gutta-percha and AH Plus sealer (Dentsply Sirona Inc., Charlotte, North Carolina, United States) (Figure 4A). The access cavity was restored with resin composite (Ivoclar Vivadent AG, Schaan, Liechtenstein) (Figure 4B). The procedure was completed without complications, and the patient was scheduled for periodic follow-up.

 Follow-up and outcomes

The procedure was completed successfully without intraoperative complications. The patient was asymptomatic and scheduled for periodic follow-up visits.

## Discussion

Guided endodontics is a rapidly advancing field that has been incorporated into various clinical applications, including tooth autotransplantation, apical microsurgical procedures, and the treatment of root canal calcifications [6,7]. A comparative study assessing access cavity preparation with and without the use of guides across operators of varying experience levels demonstrated that guided access consistently produced accurate and conservative results, independent of operator skill [8]. Another investigation observed that extended pulp space obliterations occur more frequently in maxillary teeth than in mandibular teeth, a finding that was also reflected in the present case [9]. The same study further indicated that mandibular teeth achieved a greater number of optimal precision scores when static-guided coronal access was employed.

Further comparisons between static guides, dynamic navigation systems such as Denacam (Mininavident AG, Liestal, Switzerland), and freehand access techniques revealed that static navigation yielded the highest accuracy in locating calcified or pulp stone-affected canals [10]. In contrast, freehand approaches showed greater deviations in angulation, entry, and drill tip positioning. While dynamic navigation was slightly less precise, it overcame several practical challenges associated with static guides, including restricted mouth opening, placement of rubber dams, and management of irrigation. Despite these advantages, the limitations of static-guided endodontics must be acknowledged. It is less effective in cases with restricted mouth opening, absence of a straight path through the obliterated canal, or when orthodontic appliances alter tooth positioning [11]. Moreover, the technique’s success depends on the quality of CBCT and intraoral scans, as well as the precision of guide fabrication. When canals are not visible on CBCT, guided access should not be attempted, and apical surgery may be a more suitable alternative, especially in teeth with pronounced curvature [12,13].

Several clinical recommendations have been proposed to enhance outcomes in calcified canals. CBCT remains an essential diagnostic and planning tool, while magnification and enhanced illumination through loupes or operating microscopes significantly improve canal identification [14]. The use of ultrasonic tips in combination with magnification, along with prior knowledge of root anatomy and CBCT interpretation, increases the probability of locating the canal. Nonetheless, even with these strategies, freehand access carries a higher risk of perforation or deviation in calcified teeth [3].

Guided coronal access, on the other hand, offers greater precision and conserves tooth structure, making it consistent with minimally invasive endodontic principles. With the growing number of calcified cases linked to aging, regenerative therapies, and procedures involving fiber posts, guided endodontics is expected to play a larger clinical role. This trend emphasizes the importance of adequate training and careful case selection to ensure predictable treatment outcomes.

## Conclusions

Successful management of calcified root canals requires a combination of patience, thorough knowledge of pulpal anatomy and radiographic interpretation, and the integration of modern technologies. Guided endodontic therapy has emerged as a reliable, minimally invasive approach that allows precise canal localization, conservative tooth structure preservation, and reduced treatment time. This case highlights its clinical efficacy in managing pulp canal obliteration. Future research should focus on comparative evaluation of different planning software, feasibility of various materials and designs for 3D-printed guides, and optimization of burs for access cavity preparation to further enhance the predictability and applicability of guided endodontics.
